# Interannual variability of phyto-bacterioplankton biomass and production in coastal and offshore waters of the Baltic Sea

**DOI:** 10.1007/s13280-015-0662-8

**Published:** 2015-05-28

**Authors:** Catherine Legrand, Emil Fridolfsson, Mireia Bertos-Fortis, Elin Lindehoff, Per Larsson, Jarone Pinhassi, Agneta Andersson

**Affiliations:** Centre for Ecology and Evolution in Microbial model Systems - EEMiS, Department of Biology and Environmental Science, Linnæus University, 39182 Kalmar, Sweden; Department of Ecology and Environmental Science, Umeå University, 90187 Umeå, Sweden

**Keywords:** Phytoplankton, Bacteria, Baltic Sea, Production, Climate change, Microbial foodwebs

## Abstract

The microbial part of the pelagic food web is seldom characterized in models despite its major contribution to biogeochemical cycles. In the Baltic Sea, spatial and temporal high frequency sampling over three years revealed changes in heterotrophic bacteria and phytoplankton coupling (biomass and production) related to hydrographic properties of the ecosystem. Phyto- and bacterioplankton were bottom-up driven in both coastal and offshore areas. Cold winter temperature was essential for phytoplankton to conform to the successional sequence in temperate waters. In terms of annual carbon production, the loss of the spring bloom (diatoms and dinoflagellates) after mild winters tended not to be compensated for by other taxa, not even summer cyanobacteria. These results improve our ability to project Baltic Sea ecosystem response to short- and long-term environmental changes.

**Electronic supplementary material:**

The online version of this article (doi:10.1007/s13280-015-0662-8) contains supplementary material, which is available to authorized users.

## Introduction

Projecting ecosystem structure and function in a changing climate requires a quantitative description of foodwebs with a good representation of the impact of top-down and bottom-up drivers for individual ecosystems (Heymans et al. 2014). In the Baltic Sea, recent models project an increased stress on foodwebs from changes in temperature, salinity, oxygen, and ice cover (Eilola et al. 2013; Klais et al. 2013; Carstensen et al. 2014; Meier et al. 2014), in seasonal nutrient dynamics (Arheimer et al. 2012), and in algal blooms dynamics (Klais et al. 2011, 2013; Wasmund et al. 2011; Eilola et al. 2013; Hense et al. 2013). Quantitatively, the pelagic basal component (both heterotrophic bacteria and phytoplankton) is seldom characterized in foodweb models despite its major contribution to biogeochemical cycles (photosynthesis and respiration) in marine ecosystems. There are no common trends in all sub-regions of the Baltic Sea in temporal or spatial patterns of diatoms and dinoflagellates, indicating a strong regional character of the mechanisms regulating the changes in phytoplankton dynamics (Klais et al. 2011, 2013). Community structure and ecosystem processes often vary along regional gradients, even in the absence of physical barriers. Time series analyses of the variability of phytoplankton have been done successfully in most of the Baltic Sea (HELCOM 2009), including the Baltic proper (Klais et al. 2011). In the Gulf of Bothnia, long-term microbial productivity was examined to assess the impact of climate-driven environmental change on foodweb efficiency (Wikner and Andersson 2012). In contrast, the Western Gotland Sea has been undersampled for microbial plankton abundance and production, while one of the most comprehensive datasets on fish stocks is available in this region (Kalmar Sound) (Ljunggren et al. 2010). In this region, the projects ECOCHANGE and PLANFISH have yielded extensive data, in terms of spatial and temporal coverage, hydrography, plankton ecology, biogeochemical processes, and microbial dynamics. Here we use these observations to address the influence of environmental changes and interannual variability on phyto-bacterioplankton coupling (biomass and production), and how different phytoplankton groups are related to bacteria. In addition, the spatial and temporal resolutions of the observations provide a unique comparison of coastal and offshore ecosystems.

## Materials and methods

### Study area and sampling

**Fig. 1:**
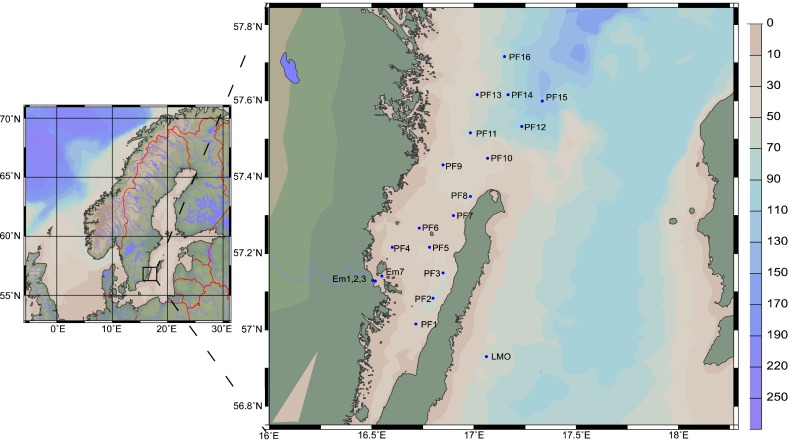
Bathymetric map of the study site and sampling stations (Ocean Data View ODV). Stations are abbreviated as *Em* Emån rivermouth area, *PF* PLANFISH stations in the Kalmar sound and the Western Gotland Sea, *LMO* Linnæus Microbial Observatory

The study area comprises approximately 100 × 100 km area from the Emån river mouth, across the northern part of the Kalmar Sound, extending to the southern part of the Western Gotland Sea, and south to the Linnæus Microbial Observatory (LMO) located approximately 11 km (6 nautical miles) off the NE coast of Öland (Fig. 1).

The study area is divided into coastal and offshore regions on the basis of bathymetry, hydrography, and ecology. The depth of coastal stations (Em1-3, Em7, PF1-4) was 1–18 and 40–150 m in offshore stations (PF11-16, LMO). All Em stations (coastal) were sampled bi-monthly during the ice-free period from April 2011 to April 2012. All PF stations (PF1-16) were sampled on a monthly basis during the productive period (April–October) over 2010–2012 during cruises aboard RV MIMER within the large-scale field experiment PLANFISH (for details, see Díaz-Gil et al. 2014). High frequency sampling (twice weekly) was carried out at LMO over 2011–2012. At each station, sampling was concentrated in the euphotic zone using a CTD probe (AAQ 1186-H, Alec Electronics, Japan) for temperature, salinity, and light profile. Water was collected with a Ruttner water sampler (5–10 L) at different discrete depths (Em), 0–10 m (PF) and at 2 m (LMO). Water was distributed in HCl washed and seawater rinsed PET bottles for nutrient and DOC analyses, and for bacterial and phytoplankton composition, biomass, and production. Analytical methods and data analysis are presented in the Supplementary Material. Daily primary production and bacterial production were integrated for the euphotic zone to allow consistent comparison between years and stations. These values were used to estimate the annual net primary and bacterial production at Em and LMO stations. For annual production, a model estimate was derived from measured daily production values extrapolated over the whole year for the period 2011–2012 (*n* = 40).

### Statistical analyses

To test the effect of temperature and nutrients on bacterial and phytoplankton biomass, we used linear models. Biomass data were transformed to fit normal distribution assumption. Due to a strong colinearity of the explanatory variables, a principal component analysis (PCA) was performed on the nutrient variables, and the scores of the first and second component were then used as explanatory variables in the models (for details see Supplementary Material). Interannual and spatial variations in biomass, relative abundance, and contribution to carbon of different phytoplankton taxa were tested using one-way ANOVA and repeated measures ANOVA with a Bonferroni post hoc test. All statistics and graphs were performed using the R software (nlme package), version 3.0.2. Marine data stations and time series plots were generated in Ocean Data View.

## Results

### Seasonality

**Fig. 2:**
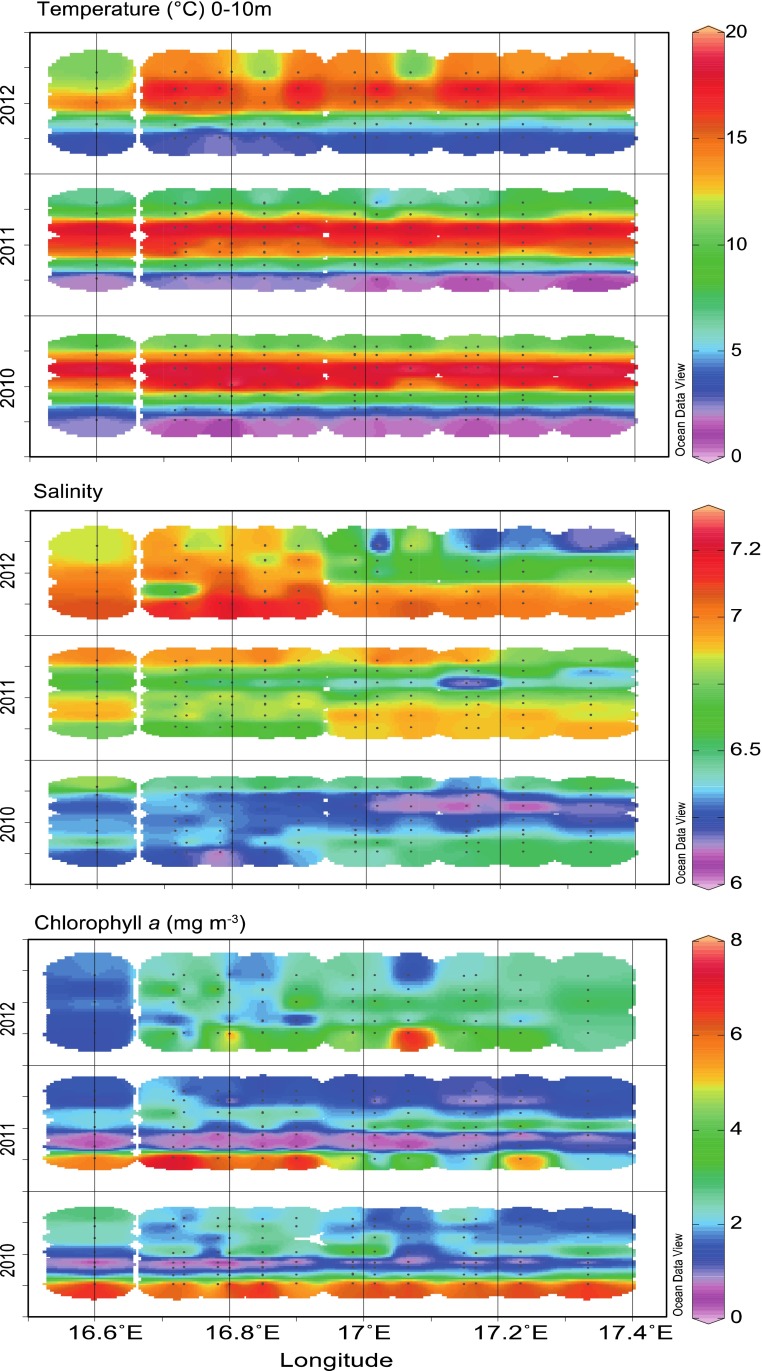
Spatial and seasonal variations in temperature, salinity, and chlorophyll a concentration in the euphotic zone (10 m) over 2010–2012 in the study area. Quarters are shown in *y axis* (ODV plots)

We present observations obtained in the Kalmar Sound and the Western Gotland Sea over three years, 2010–2012 (Fig. 2). Each year’s sampling depicts the temporal and spatial patterns of bacterioplankton and phytoplankton dynamics from the littoral zone of the Emån river estuary to the offshore waters north (>90 m depth) and east (upwelling zone, Linnæus Microbiological Observatory) of the island Öland. A similar seasonal cycle in the coastal and offshore regions enabled the comparison of the spatial and seasonal patterns in biomass and production. Overall average temperature was similar for the three years but coastal and offshore stations exhibited different hydrological characteristics (Table S1, Supplementary Material).

Our focus is in the upper 20–30 m of the water column, the euphotic zone in which most of the basal production occurs in the Baltic Sea. The year-to-year temperatures range from −0.5 to 3 °C in February–March to 20 °C in August (Fig. 2). Average sea surface temperature (SST) was similar over the three years (10.3–11.1 °C). Years 2010 and 2011 were characterized by a cool winter and strong stratification in May (Fig. 2). In 2011, the second half of the year was warm and led to a mild winter 2012. Only 4 days were below 3 °C in 2012 compared to more than 100 in 2010 and 2011 (Table S2). Deep mixing occurred from February to June 2012 leading to late stratification in the study area (Fig. S1). Inflow of polar air masses and storms in summer 2012 contributed to a cool summer (Siegel and Gerth 2013), and SST was below 20 °C from June to August (Fig. 2). Surface salinity ranged from 6 to 6.8 in 2010–2011 to over 7.2 in 2012 (Fig. 2, Table S1) and there was no strong stratification till June 2012. The surface distribution of inorganic nutrients and total nitrogen and phosphorus showed considerable scatter due to both seasonality and locations (coastal, offshore) (Table S1). Nitrate, silica, total N, and DOC were significantly higher in coastal regions influenced by the Emån estuary. High phosphate (>2 µM) was traced in late spring and summer offshore (LMO) indicating upwelling events in 2011 and 2012.

### Phytoplankton dynamics and community structure

We observed a high interannual variability in chlorophyll *a* between 2010–2011 and 2012 (Fig. 2). Intense spring blooms occurred in 2010 and 2011 (cool winter) and vanished fast as 7–8 mg chlorophyll *a* m^−3^ disappeared from the water column in approximately 1 month (Fig. 2). Overall average phytoplankton biomass was higher in 2010, where all taxa except for small flagellates contributed >20 % (Table 1). Diatoms and dinoflagellates dominated phytoplankton communities in well-mixed coastal and offshore waters during the spring in 2010 and 2011 contributing up to 58 and 44 % to the total phytoplankton biomass (Fig. S2, Table 1). The diatom community was diverse but dominated by chain-forming pelagic species (*Chaetoceros*, *Skeletonema,* and *Thalassiosira*). With stratification of the water in early May, summer blooms peaked at 4 mg chlorophyll *a* m^−3^, while smaller autumn blooms lasted till October in 2010 and 2011. Filamentous cyanobacteria were present since April and peaked in July–August. They accounted for 12–34 % of the annual phytoplankton biomass with maximum (>95 %) in the summer.

**Table 1:** Relative contribution of phytoplankton taxa to the stock biomass over 2010 and 2011 (cool winter), and 2012 (mild winter) for the Kalmar Sound (mean values for stations PF1-16, *n* = 318). Statistical significance of interannual variation of phytoplankton biomass and composition (taxa contribution) (Repeated measures ANOVA with Bonferroni post hoc test). *** *p* < 0.001; ** *p* < 0.01; * *p* < 0.05

	2010	2011	2012
Phytoplankton biomass (mg C m^−3^)	168***	118	96
Diatoms (mg C m^−3^) (%)	49 (29)	68 (57.6)	3 (3)***
Dinoflagellates (m C m^−3^) (%)	74 (44)	26 (22)*	48 (50)
Small flagellates (mg C m^−3^) (%)**	8 (5)***	13 (11)	21 (22)
Cyanobacteria (mg C m^−3^) (%)*	34 (20)	12 (10)	23 (24)

In 2012 (following a mild winter), the spring bloom was lower (3–4 mg chlorophyll *a* m^−3^) than in the previous years and comparable to summer or autumn blooms in intensity. In 2012, diatoms were only minute contributors to the community (<5 %), while dinoflagellates and small flagellates made up most of the biomass (>70 %). The diatom blooms were mostly monospecific (either *Chaetoceros* or *Skeletonema*). Following cool summer temperature and stratification, filamentous and colonial cyanobacteria were present till late autumn.

### Environmental drivers and bacteria–phytoplankton coupling

The linear regression modeled both bacteria and phytoplankton biomass by selecting temperature, and phosphate–nitrate–total phosphorus (pc1) as the most relevant predictor variables (Table 2). Pc1 made the greatest contribution to the models for both bacteria and phytoplankton. For phytoplankton only, the models selected significant interactions between temperature and nutrients (pc1), with high biomass at low temperature (spring bloom) and low nutrients (summer blooms). The overall *r*^2^ for the models were 0.851 for bacteria and 0.575 for phytoplankton.

**Table 2:** Linear regression analyses for bacteria and phytoplankton biomass, and temperature and nutrients. Variables included in pc1: phosphate, total phosphorus, nitrate; in pc2: silica and ammonium. For data transformations see details in Supplementary Material. N.S. non-significant

Bacterial biomass					Phytoplankton biomass			
Parameter	Estimate	SE	*t* value	*p*	Estimate	SE	*t* value	*p*
β_0(Intercept)_	11.517	1.066	10.799	<0.001	1.708	0.084	20.376	<0.001
β_Temperature_	−1.566	0.202	−7.761	<0.001	−0.008	0.016	−0.526	N.S
β_Temperature_^2^	0.085	0.009	9.060	<0.001	0.0001	0.0007	0.194	N.S
β_pc1_	1.135	0.403	2.820	<0.01	0.075	0.032	2.355	<0.05
β_pc2_	0.142	0.444	0.319	N.S	−0.092	0.035	−2.635	<0.05
Interactions								
β_Temperature * pc1_	−0.109	0.104	−1.055	N.S	−0.022	0.008	−2.664	<0.01
β_Temperature_^2^_* pc1_	0.003	0.006	0.447	N.S	0.001	0.0004	2.822	<0.01
β_Temperature * pc2_	0.025	0.120	0.212	N.S	0.0007	0.009	0.073	N.S
β_Temperature_^2^_* pc2_	−0.002	0.006	−0.385	N.S	0.0004	0.0005	0.818	N.S
Model summary	Adjusted *r* ^2^ = 0.851		*p* < 0.001		Adjusted *r* ^2^ = 0.575		*p* < 0.001	

**Fig. 3:**
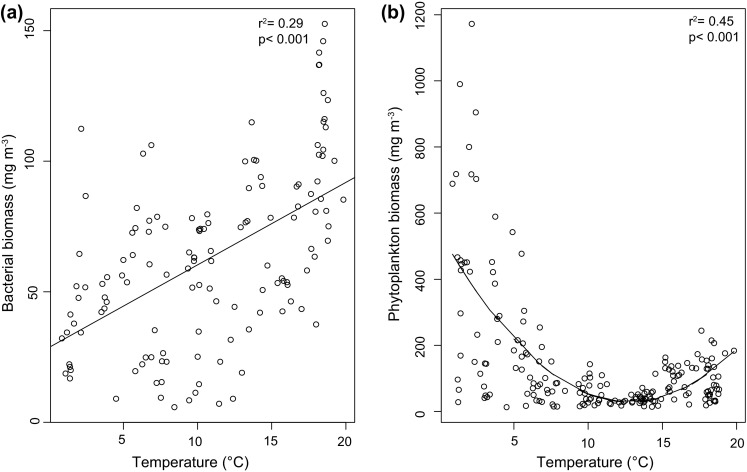
Relationship between bacterial (**a**) and phytoplankton biomass (**b**) with temperature during 2010–2012 in the study area (*n* = 195). Bacterial biomass was positively associated to temperature (linear regression, *r* ^2^ = 0.29, *p* < 0.001) while phytoplankton biomass was mainly affected by temperature <10 °C (polynomial regression, *r* ^2^ = 0.45, *p* < 0.001) over the three years

Bacterial biomass was positively correlated with temperature (slope 3.162, *r*^2^ = 0.29, *p* < 0.001) (Fig. 3). The unimodal relationship between phytoplankton biomass and temperature (*β*_1_ = −80.1840, *β*_2_ = 3.1270, *r*^2^ = 0.45, *p* < 0.001) (Fig. 3) was mostly driven by the negative association of diatoms with temperature (*F*_2,464_ = 86.83, *β*_1_ = −41.286, *β*_2_ = 1.63, *r*^2^ = 0.236, *p* < 0.001, data not shown) and a weak positive association of cyanobacteria (*F*_2,463_ = 86.76, *β*_1_ = −4.33, *β*_2_ = 0.43, *r*^2^ = 0.269, *p* < 0.001, data not shown). Overall there was no significant temporal coupling between bacteria and phytoplankton biomass (*F*_1,351_ = 0.629, *r*^2^ = 0.001, *p* = 0.428) nor production (*F*_1,44_ = 1.481, *r*^2^ = 0.032, *p* = 0.23).

### Coastal–offshore relationships and trends

**Fig. 4:**
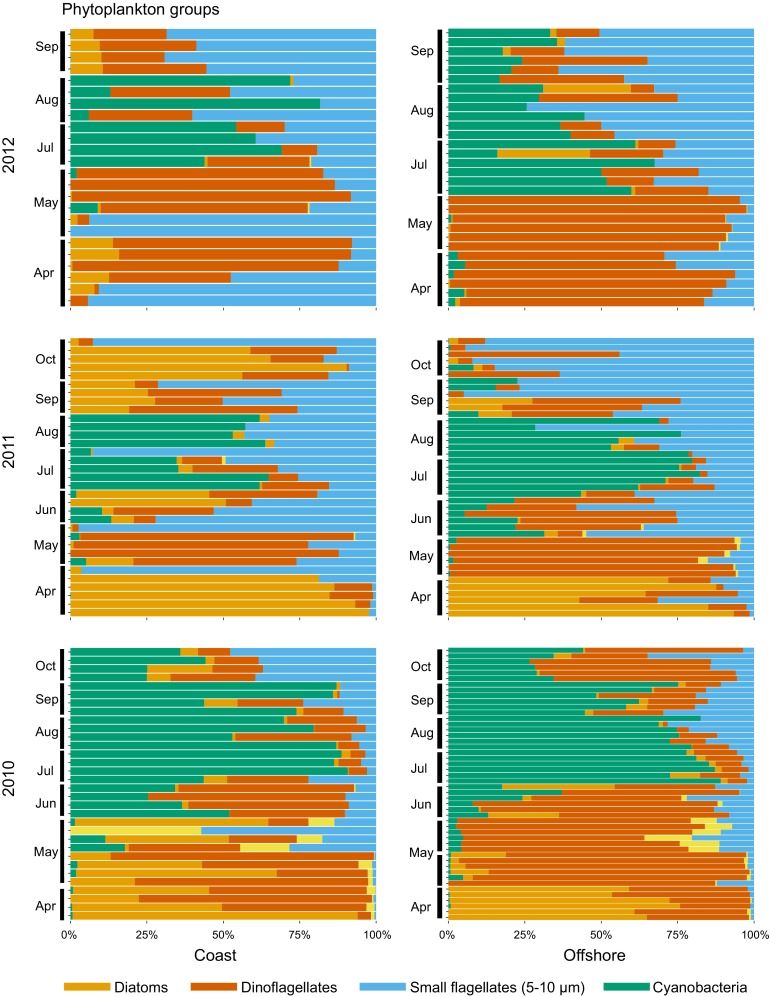
Relative abundance of selected phytoplankton taxa to total phytoplankton biomass in selected coastal and offshore stations over the period 2010–2012. Each bar is a discrete sample (0–10 m) at coastal (Em1-3, Em7, PF1-4) and offshore (PF11-16, LMO) stations

Interannual variations were high between the three years and there were no significant differences in bacterial and phytoplankton biomass between coastal and offshore areas for the three years (Fig. S3a, b). On an annual basis, bacterial stock biomass was higher in coastal than offshore areas (Table 3). Annual cumulative phytoplankton biomass was lower in coastal (19.3 g C m^−3^) than in offshore (28.4 g C m^−3^) areas, but no significant differences over the range. Annual primary and bacterial production estimates encompassed a broader range in coastal areas than offshore (Table 3). In coastal areas, our model provided a broad range for primary (tenfold) and bacteria (30-fold) production. Despite these large variations, coupled to hydrographic conditions, the similarities in trend of increased bacterial and primary production from coastal (Emån estuary stations) to offshore (LMO) areas illustrate coupling on a spatial scale (Fig. S3c, d). Coastal phytoplankton community seasonal patterns were similar to those observed offshore with a dominance of dinoflagellates in the spring bloom 2012 (mild winter, late stratification) (Fig. 4). Overall in coastal areas, diatoms and dinoflagellates contributed a similar amount (21–24 %) to the annual phytoplankton stock biomass (Table 3). Coastal areas were characterized by a significant contribution of diatoms and small flagellates to the annual phytoplankton stock biomass (Table 3). Offshore areas were defined by a significantly higher contribution of filamentous and colonial cyanobacteria to stock biomass.

**Table 3:** Annual bacterial and phytoplankton biomass and production in coastal and offshore areas and relative contribution of phytoplankton taxa to the stock biomass over the productive period February–October. Average (min–max) values of biomass for coastal (Em1-3, Em7, PF1-4) and offshore (PF11-16, LMO) stations are given. For annual production, measured daily production values were extrapolated over the whole year for the period 2011–2012. Statistical significance (one-way ANOVA) is *** *p* < 0.001, ** *p* < 0.01, * *p* < 0.05

	Coast	Offshore
Phytoplankton biomass (g C m^−3^)	19.3 (14.1–33.2)	28.4 (16.6–57.5)
Bacterial biomass (g C m^−3^)**	15.8 (13–19.8)	11.9 (11–12.5)
Primary production (g C m^−2^ year^−1^)	142.5 (32.7–257.5)	378.5
Bacterial production (g C m^−2^ year^−1^)	29.5 (3.8–59.4)	33.4
Annual contribution (mean %, min–max)		
Diatoms***	24 (0–71)	6 (0–62)
Dinoflagellates	21 (1–52)	33 (0–88)
Small flagellates*	42 (7–97)	26 (1–80)
Cyano*	13 (0–41)	35 (0–99)

## Discussion

### Seasonality and diatoms–dinoflagellates interactions in “mild winter” years

Overall, the phytoplankton succession in the Kalmar Sound and the Western Gotland Sea consisted of a spring bloom with diatoms and dinoflagellates (April–May) and a long summer bloom dominated by filamentous cyanobacteria and a mix of small flagellates and dinoflagellates (June–October). Dinoflagellates dominated primary production together with small flagellates until the summer cyanobacterial blooms. The phytoplankton biomass was the highest during “cool winter” compared to “mild winter” years. Our results also highlight that in terms of annual biomass there was no compensation for the low contribution of diatoms to the spring bloom 2012.

Diatoms are an essential part of the Baltic Sea foodweb, often prevailing over the other primary producers on an annual basis (Wasmund et al. 2011). Spring blooms in the Baltic Sea are not typical ocean diatom-dominated blooms, since diatoms and cold-water dinoflagellates co-exist prior to stratification of the water column (Wasmund and Uhlig 2003). A decline of diatom contribution to the spring bloom has been documented in the Baltic Proper and the Gulf of Finland over the last four decades (Klais et al. 2011; Wasmund et al. 2011), especially after mild winters (Wasmund et al. 2013). This shift in spring bloom composition has implications for the biogeochemistry of the different basins, nutrient cycling, and the foodweb efficiency of coastal and offshore regions. Diatoms sink fast to the bottom with their siliceous frustules and play a major role in pelagic–benthic coupling, while dinoflagellates production is primarily recycled in the water column (Höglander et al. 2004).

Recent evidence points out that hydrography (Wasmund and Uhlig 2003), potential silica limitation (Danielsson et al. 2008), and climate change effects (Höglander et al. 2004; Klais et al. 2011, 2013) shape the spring phytoplankton communities in the Baltic Sea. In the Western Gotland Sea and the Kalmar Sound, the proportion of diatoms to the annual phytoplankton biomass decreased by ten to 20-fold between 2010 and 2011 and 2012, while dinoflagellates were present in the same range. A common perception is that turbulence and late stratification of the water column favor diatoms (Margalef 1978). Wasmund and Uhlig (2003) hypothesized that early stratification caused by warmer temperatures could cause diatoms to sink out of the euphotic zone and favor dinoflagellates. However, mixing events have been numerous during the study period; with strong upwellings toward the Swedish east coast in 2011, storms in 2012 (Siegel and Gerth 2013) and a large inflow of saline water from the Kattegat to the Baltic Sea in December 2011 (Nausch et al. 2013). Recent evidence showed that ambient wind mixing does not benefit diatoms (Klais et al. 2013), and that the relative biomass of diatoms can be low despite late stratification in early summer (this study). Our data emphasize that turbulence and stratification may not be the crucial causal mechanism for the contribution of diatoms to the spring bloom in the Western Gotland Sea.

Nutrient levels in the Baltic Sea are influenced by nutrient supply from land and benthic–pelagic fluxes (Eilola et al. 2009). We found that nutrient conditions after ice breakup were influenced by the Emån river discharge (coastal stations) and upwelling of nutrient-rich deepwater (coastal and offshore). There was no direct evidence of silica-limiting diatom growth, despite spatial variability of silica levels in the study area.

Time series data collected at LMO (offshore) revealed that dinoflagellates were already present in February (data not shown). Many dinoflagellates form resting cysts at the end of the growth period and excyst upon favorable conditions (Kremp 2013). The winter 2012 was “mild” (4 days below 3 °C), with no or thin ice cover, and could have lead to early excystment. The dominance of cold-water dinoflagellates in the spring bloom depends to a large extent on the size of the initial population (Kremp 2013), and could explain their large dominance till early summer in the Western Gotland Sea. Temporal series from the Northern Baltic Sea (30 years, Klais et al. 2013), the Southern Baltic Sea (22 years, Wasmund et al. 2013), and high spatial coverage in the Western Gotland Sea (3 years, this study) point out that in a warmer Baltic Sea, with thinner and shorter lasting ice, dinoflagellate spring communities will be favored over diatoms in spite of vertical mixing and late stratification. However, the causal mechanisms are still circumstantial. Inflow of saline water mainly affects deepwater variability, and the benthic–pelagic coupling can explain high salinity, late stratification, and cool SST in summer 2012; hydrographic conditions in which dinoflagellates thrived. The future Baltic Proper has been projected to be warmer and fresher (Meier et al. 2014). The effect of climate-driven changes in salinity should not be underestimated in future models since a drop in salinity may have antagonistic interactions with the development of dinoflagellates.

### Climate-hydrological factors affect phytoplankton and bacteria coupling

A strong coupling between phytoplankton and bacteria was not expected in the study area since bacteria rely little on phytoplankton DOC in areas under the influence of coastal inputs of DOC. The share of terrestrial dissolved organic matter (DOM) present in the open Baltic Sea is >50 % and the contribution from phytoplankton release is minor (Deutsch et al. 2012). Bioavailability of terrestrially derived DOM depends on the different land uses in the catchment area. The large influence of agricultural land use in the Kalmar Sound area means that terrestrial DOM is likely to be transported to both coastal and offshore stations, relatively unaffected by heterotrophic processes near shore (Asmala et al. 2013). In the study area, pigmented cells dominate among nanoflagellates (75–90 %, Sopanen et al. 2009). The lack of correlations between bacteria and total nanoflagellates and between bacteria and ciliates (data not shown) suggests no tight control from predation. This supports that bacterial production is subject to bottom-up control by DOM in high-productivity waters, more than predation (Gasol et al. 2002). This apparent uncoupling between heterotrophic bacteria and primary producers over the whole study area also highlights the importance of identifying the boundary between coastal and offshore areas in the Baltic Proper in relation to terrestrial DOM runoff, and to what degree this would be affected by future changes in precipitation patterns.

### The role of temperature

While nutrient limitation drives primary production in the stratified euphotic zone, temperature affects metabolic rates of both photosynthesis and respiration and thus indirectly primary and secondary production, e.g., the ratio of autotrophy and heterotrophy. Mesocosm experiments gave evidence that rising temperature alters bacterial community composition (Lindh et al. 2013) and drives microbial foodwebs toward heterotrophy (von Scheibner et al. 2014). In polar regions, bacterial production is regulated indirectly by sea ice cover and its impact on primary production (Ducklow et al. 2012). An inverse foodweb model confirmed that herbivore–diatom-dominated foodwebs are replaced by microzooplankton–small phytoplankton–bacteria foodwebs in relation to temperature (Sailley et al. 2013). In the Western Gotland Sea, heterotrophic bacteria communities were reasonably predicted by temperature on an annual basis over the 3-year study for temperature below 20 °C (Fig. 3). While causality based on short-term time series should not be overinterpreted, our findings confirm that bacterial biomass could be used as a key indicator of climate variability (Morán et al. 2010).

Metabolic theory predicts that enhanced metabolism in unicellular organisms will result in lower biomass at higher temperature (Brown et al. 2004). This prediction, that phytoplankton will decrease with increasing temperature in the ocean, has been projected in the global ocean (IPCC 2013) and confirmed in the Atlantic Ocean (Morán et al. 2010). In the Western Gotland Sea, temperature affected the abundance and biomass of phytoplankton in a different way and will likely alter the trophic relationship with heterotrophic bacteria. The negative relationship between phytoplankton biomass and temperature >8 °C suggests a decrease of the spring bloom production in the future Baltic Sea, regardless of the cold-water diatom–dinoflagellates ratio.

However, projections diverge since algal physiological responses predict that phytoplankton including cyanobacteria should grow faster in warmer waters (Paerl and Huisman 2008; Hense et al. 2013), especially in nutrient-rich systems. Eco-evolutionary models project that optimal temperature for cold-water phytoplankton strains should increase with mean seawater temperature (local adaptation), and exceed it by 3°–5° (Thomas et al. 2012). This margin is in the range of projected rising temperature of the Baltic Proper basin (Meier et al. 2014). Therefore, at high nutrient loads (spring), rising temperature may increase primary production but shift the community to smaller cells (more nanoplankton and bacteria).

Combining the bacteria–phytoplankton temperature response (Fig. 3) with the projected 3–5 °C SST increase, we hypothesize that in the 8–14 °C temperature window, the ratio between autotrophy and heterotrophy (1–2:1) will drive toward a heterotrophic system at the basal trophic levels. Despite the expected decline of labile DOC toward the open sea, bacteria were constrained first by temperature, and then by nutrients (this study). “Heterotrophy” would have to include small flagellates and dinoflagellates as many pigmented forms are mixotrophic (Flynn et al. 2013) and they will likely become dominant in microbial pelagic communities in the future Baltic Sea. Climate change is a potent driver for both picocyanobacteria and filamentous cyanobacteria bloom expansion (Paerl and Huisman 2008). In the 15–22 °C temperature window, the ratio between autotrophy and heterotrophy could shift back toward autotrophy dominated by prokaryotes. However, the annual biomass of cyanobacteria would still be lower (250 mg C m^−3^) than the spring bloom (600–1000 mg C m^−3^) according to our results, provided unchanged grazing pressure. In the Baltic Sea, coupled climate models integrating plankton physiological responses in their projections (Hense et al. 2013) are needed. There is a substantial genetic variability in bloom populations (Kremp 2013), thus the success of one given genotype (phytoplankton or bacteria) due to changes in ocean climate may be dependent of the strains present in the region.

### Coastal–offshore interactions

Annual primary production in the Baltic Sea is generally higher in an average river plume in comparison to open sea areas (Wasmund et al. 2001). Increasing distance from the coast toward the open sea is usually associated with a decline in terrestrial organic matter, nutrients, temperature, and increasing salinity. In the Western Gotland Sea, DOC, nitrogen, and silica levels decreased from coast to offshore areas, but phosphate enrichment of the upper mixed layer was detected in offshore stations during late spring and summer time after stratification. Upwelling is among the most important mechanisms causing vertical mixing and deepwater intrusions in both offshore and coastal areas of the Baltic Sea (Myrberg and Andrejev 2003). Upwelling areas are relatively frequent on the whole offshore coast (<50 km) of the Western Baltic Proper, which together with dominant westerly winds in 2011–2012 and the North Sea inflow in December 2011 could explain the enhanced primary productivity at LMO (350 g C m^−2^ year^−1^) compared to Emån river estuary (26–260 g C m^−2^ year^−1^).

On the basis of annual primary production (in situ), both coastal and offshore areas in the Kalmar Sound and Western Gotland Sea can definitely be characterized as “eutrophic” according to Wasmund et al. (2001) and the Baltic Sea Action Plan status classification (HELCOM 2009). Primary production has roughly doubled (100–200 g C m^−2^ year^−1^) during the past century due to eutrophication in the Baltic Proper (Elmgren 1989), and can reach up to 400 g C m^−2^ year^−1^ (this study). These results support that averaging across large geographic areas can lead to an underestimation of the trends in ecosystem response to environmental drivers (Ducklow et al. 2012). Further, it raises the question as to whether the ecosystem response of the Kalmar Sound and the Western Gotland Sea to eutrophication and rising temperature is linear considering that phytoplankton community composition may have changed as it did during the past four decades in the Gulf of Finland and the Southern Baltic (Wasmund et al. 2011; Klais et al. 2011).

Anthropogenic disturbances and climate change can strongly influence trophic cascades through marine foodwebs in littoral/coastal and in offshore/pelagic areas (Casini et al. 2008; Eriksson et al. 2011). In their Baltic Sea model, Casini et al. (2008) showed evidence that phytoplankton variation was solely explained by top-down processes using chlorophyll data from the Gotland basin over 1974–2006. This is obviously not the case in the current study. The limited number of observations of microbial composition and production used in ecological models may have an impact on the interpretation of trends in time series. In the light of our findings, biomass could be a better indicator than chlorophyll *a* to project plankton dynamics in foodweb models in aquatic systems dominated by small flagellates.

## Conclusions

Our current understanding of foodweb dynamics may challenge our ability to project the response of foodwebs to changing climate. We propose that planktonic microbial communities merit particular attention to understand better how communities and ecosystem respond to changing climate. Our results show that interannual and regional differences in phyto- and bacterioplankton reflect changes in temperature, nutrients, and salinity in the Western Gotland Sea. Our high spatial and temporal resolution dataset adds to the conclusions of empirical studies that the spring bloom is reduced during mild winters. In terms of annual carbon, the loss of the spring bloom (diatoms and dinoflagellates) tends not to be compensated by other taxa. In the long run, this may reduce the total microbial production transfer to higher trophic levels.

## Electronic supplementary material

Supplementary material 1 (PDF 5112 kb)
