# Supplementary material for: Interannual variability of phyto-bacterioplankton biomass and production in coastal and offshore waters of the Baltic Sea
Source: Ambio. 2015 May 28;44(Suppl 3):427–38. doi: 10.1007/s13280-015-0662-8 (PMC4447688; doi:10.1007/s13280-015-0662-8)
Supplement: Supplementary file 1 — Supplementary material 1 (PDF 5112 kb) [file 13280_2015_662_MOESM1_ESM.pdf]

AMBIO

Electronic Supplementary Material

This supplementary material has not been copy edited by the publisher or the editorial office.

Title: **Interannual variability of phyto-bacterioplankton biomass and production in coastal and offshore waters of the Baltic Sea**

Authors: Catherine Legrand, Emil Fridolfsson, Mireia Bertos-Fortis, Elin Lindehoff, Per Larsson, Jarone Pinhassi, Agneta Andersson

Analytical methods

Chlorophyll *a*, bacterial abundance, phytoplankton abundance and composition were determined at every station. Chlorophyll *a* was measured fluorometrically on ethanol extracts. Samples for bacterial abundance were preserved in 2% formaldehyde, kept at -80°C and analyzed using flow cytometry (BDFACs Calibur) using SYTO13 (Gasol and del Giorgio 2000). Samples for phytoplankton abundance were preserved with 2% Lugol's solution, kept in the dark and transferred into sedimentation chambers. Cells were counted using an Olympus CK X41 microscope. At least 300 cells were counted in each sample. Cells were identified to the genus or species level when possible. Nutrients were measured using a UV-1600 spectrophotometer (VWR) as described in (Valderrama 1995*)*. Dissolved organic carbon (DOC) and total nitrogen (Tot-N) were measured simultaneously via high temperature catalytic oxidation (HTCO) using a Shimadzu TOC-V analyzer coupled to a TNM-1 unit (Pages and Gadel 1990).

Primary production was measured at Em and LMO stations by ^{14}C bicarbonate incorporation (specific activity 25 µCi ml^{-1}, 0.075 µCi ml^{-1} final concentration) in 4h in–situ incubation at different depths in the euphotic zone (1% of the surface irradiance) with dark controls as described in the HELCOM guidelines (HELCOM Combine 2014). After acidification (6M HCl), samples were aerated prior to liquid scintillation counting in ScintiSafe 3 cocktail with a scintillation counter (Wallac 1414 WinSpectral Liquid Scintillation Counter).

Bacterial production was derived from ^{3}H-leucine incorporation with a protocol modified from (Smith and Azam 1992) in samples from the euphotic zone from the same depths as for primary production. Four replicates (1.2 ml) and two blanks were

incubated in the dark for two hours with ^{3}H-leucine (40 nM final concentration). Incubations were done on-deck close to in situ temperature (water bath) and terminated by the addition of 120 µl of 50% trichloroacetic acid (TCA). Samples were stored on dry ice during transport to the lab. Samples were concentrated by centrifugation (12 000 g for 10 minutes) and rinsed with 5% TCA. The procedure was repeated prior to liquid scintillation counting in ScintiSafe 3 cocktail with a scintillation counter.

Data analysis

Phytoplankton biomass was calculated as described by HELCOM Combine (2014). Bacterial biomass was converted to carbon biomass using a factor of 20 fg C cell^{-1} (Lee and Fuhrman 1987). ^{14}C uptake rates were converted to daily primary production (Steele 1962). Light attenuation coefficients derived from Secchi depths (Em stations) and CTD data (Em, PF, LMO) were used in integrating productions for the euphotic zone. Light irradiance at the water surface was extracted from the STRÅNG database. Leucine incorporation rates were converted to bacterial carbon production (0.86, Simon and Azam 1989). Daily primary production and bacterial production were integrated for the euphotic zone to allow consistent comparison between years and stations. These values were used to estimate the annual net primary and bacterial production at Em and LMO stations. For annual production, a model estimate was derived from measured daily production values extrapolated over the whole year for the period 2011–2012 (n = 40).

Statistical analysis (Table 3, data transformation prior to linear regression analyses)

Results presented in Table 3 were obtained after transformation of the response variables (bacteria and phytoplankton biomass in 2011) to fit normality assumption. Square root transformation was used for bacterial biomass, whereas Box-Cox transformation was preferred for phytoplankton biomass. Since the relationship between biomass and temperature was quadratic, a 2nd order polynomial function of temperature was included in the model. Nutrient concentrations were summarized by two integrative index derived from the scores a PCA on phosphate, nitrate, total P, total N, silicate and ammonium. The two first principal components accounted for 60% of the variance and were retained as distinct integrative nutrient variables. After a rotation of these two first PCA axes (with procedure *varimax* of package *stats*), two easily interpretable integrative variables were obtained. The first PCA component (pc1) reflected phosphate, nitrate and total P concentrations. The second PCA component (pc2) reflected the silicate and ammonium concentrations. For both components, positive values indicated high concentrations of nutrients.

References

Gasol, J. M., and P. A. del Giorgio. 2000. Using flow cytometry for counting natural planktonic bacteria and understanding the structure of planktonic bacterial communities. *Scientia Marina* 64: 197–224.

HELCOM Combine. 2014. Manual for marine monitoring in the COMBINE Programme of HELCOM. Retrieved 28 May, 2014, from http://helcom.fi/action-areas/monitoring-and-assessment/manuals-and-guidelines/combine-manual

Kowalewski, M. 2005. The influence of the Hel upwelling (Baltic Sea) on nutrient concentrations and primary production – the results of an ecohydrodynamic model. *Oceanologia* 47: 567–590.

Lee, S., and J. A. Fuhrman. 1987. Relationships between biovolume and biomass of naturally derived marine bacterioplankton. *Applied and Environmental Microbiology* 53: 1298–1303.

Pages, J., and F. Gadel. 1990. Dissolved organic matter and UV absorption in a tropical hyperhaline estuary. *Science of The Total Environment* 99: 173–204.

Simon, M., and F. Azam. 1989. Protein content and protein synthesis rates of planktonic marine bacteria. *Marine Ecology Progress Series* 51: 201–213.

Smith, D. C., and F. Azam. 1992. A simple, economical method for measuring bacterial protein synthesis rates in seawater using ^{3}H-leucine. *Marine Microbial Food Webs* 6: 107–114.

Steele, J. H. 1962. Environmental control of photosynthesis in the sea. *Limnology and Oceanography* 7: 137–150.

Valderrama, J. C. 1995. Methods of nutrient analysis. In *Manual of Harmful Marine Microalgae. IOC Manuals and Guides, vol. 33*, ed. G. M. Hallegraeff, D. M. Anderson, and A. D. Cembella, 251–268. Paris: UNESCO.

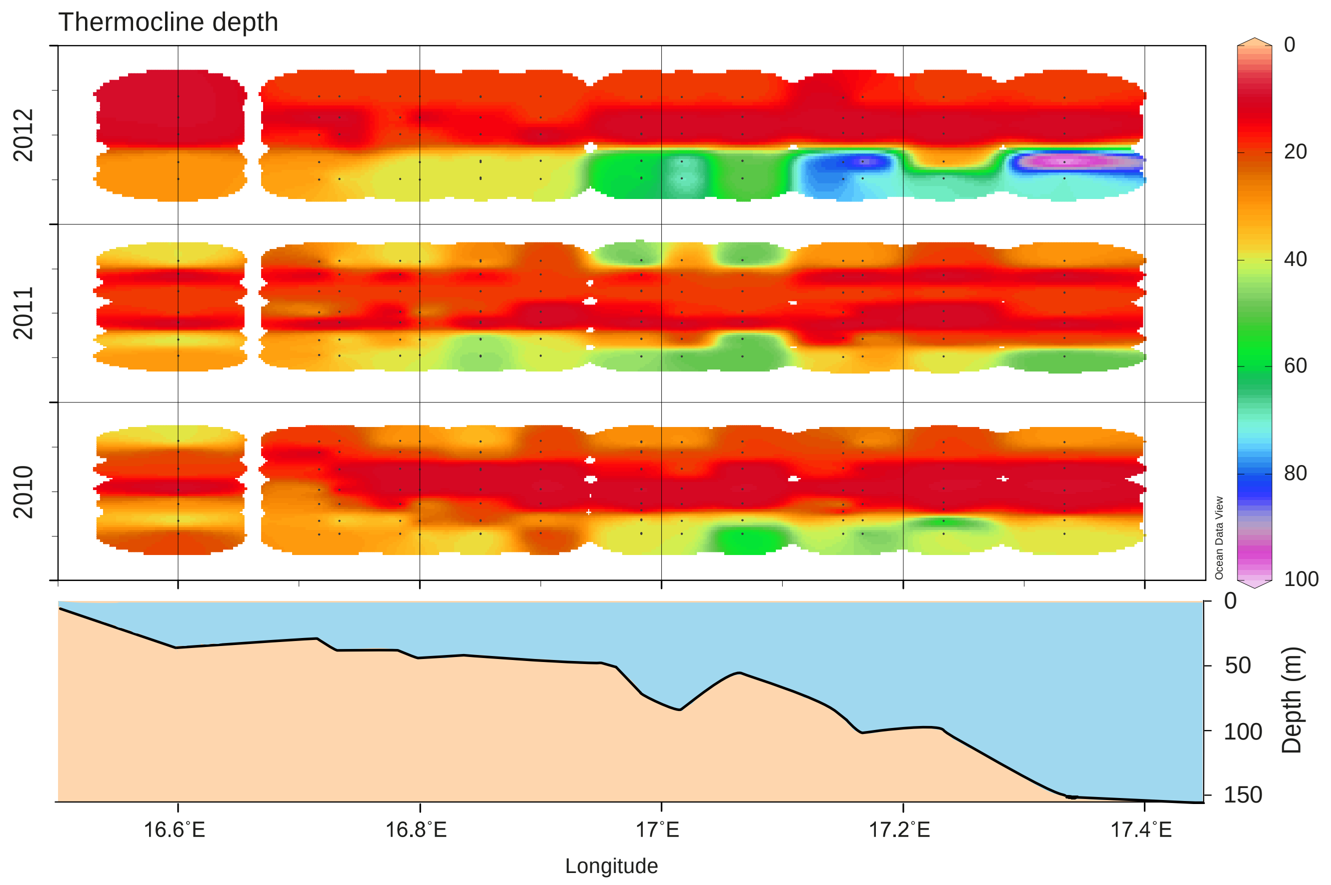

Fig. S1 Ocean Data View (ODV) plots indicating the depth of the thermocline and seafloor topography during the study period 2010-2012. Quarters are shown on y-axis

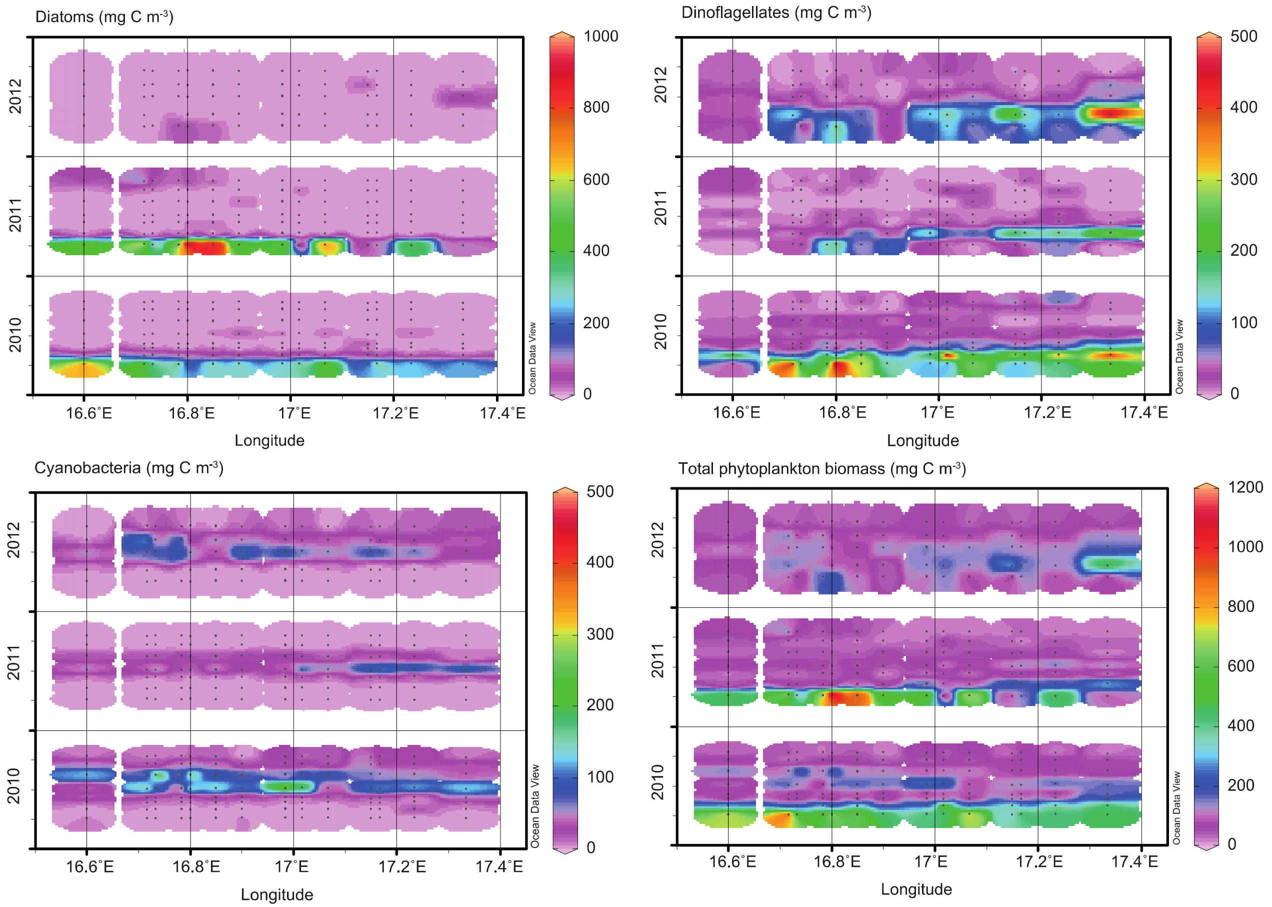

Fig. S2 Biomass of selected phytoplankton taxa and total phytoplankton in the study area over 2010-2012. Quarters are shown on y-axis (ODV plots)

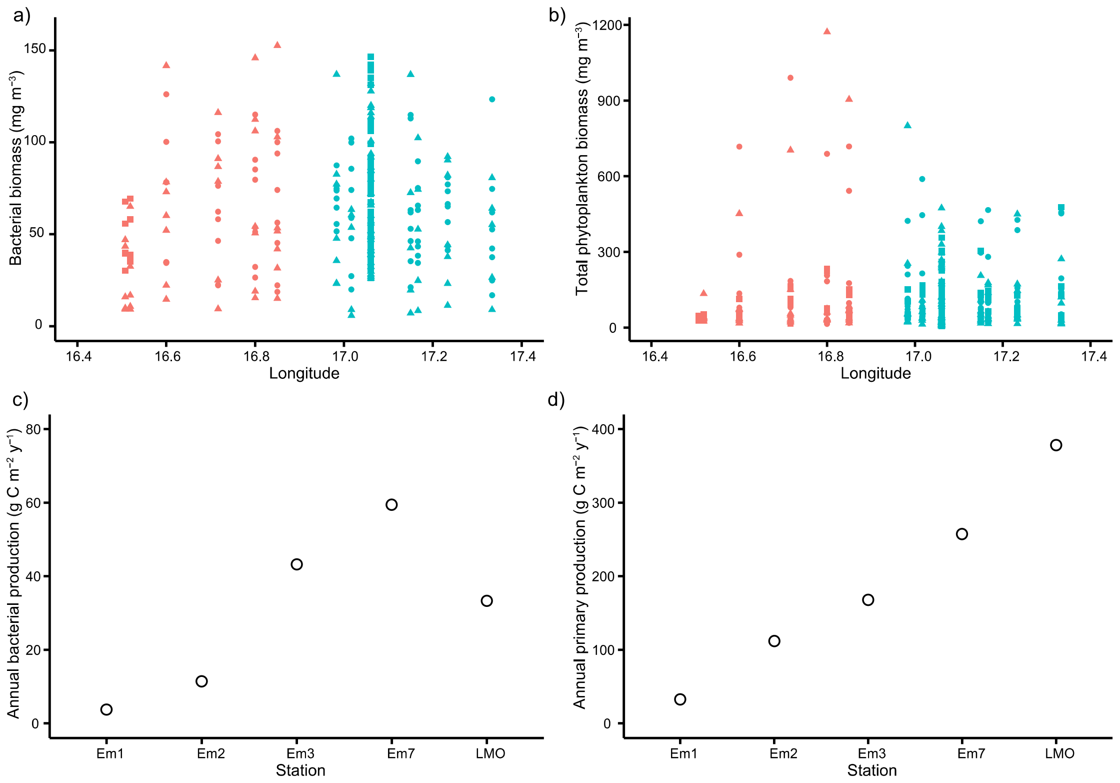

Fig. S3 Patterns of bacterial (a) and phytoplankton biomass (b) and annual production (c, d) in coastal (red) and offshore (blue) stations. No difference in biomass was found between coastal and offshore for the 3 years separately (t-test, $p > 0.05$). Dots represent data points for each year 2010 (filled diamond), 2011 (filled triangle), 2012 (filled square). Annual production (hollow circle) integrated over the productive period (February 2011-October 2012) and the euphotic zone was higher in offshore than in coastal stations

Table S1 Hydrological data for all stations (Total, n = 21), coastal (Em1-3, Em7, PF1-4; n = 8) and offshore (PF11-16, LMO; n = 7) stations over 2011-2012 in the study area. Samples were taken at 0-10 m. Maximum phosphate values in 2011 measured offshore coincide with the North Sea inflow in the Baltic Sea over December 2011. Statistical significance of spatial variation (coastal-offshore) of hydrological parameters (One-way ANOVA). *** $p < 0.001$, ** $p < 0.01$, * $p < 0.05$. Silica consumption ranged 25–50% with residual levels >5 μM. In upwelling zone, the load of nutrients from deepwater can match river input to coastal zones (Kowalewski 2005), and nutrient limitation at LMO was not likely reducing community growth rate.

	2010 mean (min-max)	2011 mean (min-max)	2012 mean (min-max)	Coastal mean (min-max)	Offshore mean (min-max)
				Period 2011-2012	
Temperature (°C)	10.66 (0.86-19.83)	10.69 (1.21-20.0)	9.80 (0.30-18.40)	9.58 (0.86-20)	10.55 (0.3-18.83)
Salinity	6.33 (6.03-6.82)	5.80 (0-7.03)	6.46 (0-7.5)	6.18 (0-7.5) ***	6.47 (5.8-7.05)
Chlorophyll a (mg m^{-3})	2.34 (0.24-7.16)	2.04 (0.23-13.45)	2.23 (0-6.84)	2.23 (0.3-11.3)	2.12 (0.23-13.45)
Nitrate (μM)	n.d.	0.75 (0-35.64)	2.93 (0.08-30.9)	4.16 (0-35.6)***	0.52 (0-3.28)
Phosphate (μM)	n.d.	0.27 (0-2.81)	0.43 (0.03-1.47)	0.36 (0.05-1.05)	0.31 (0-2.81)

Silicate (µM)	n.d.	9.64 (0-123.4)	18.40 (3.5-122.2)	20.9 (1.12-123.4) ***	10.04 (0.02-38.9)
Ammonium (µM)	n.d.	0.75 (0-3.86)	1.11 (0-3.25)	1.06 (0-3.86) *	0.79 (0-3.25)
Total N (µM)	n.d.	17.57 (6.2-62.2)	20.15 (12.8-69.44)	22.45 (0-69.5) ***	17.17 (7.21-31.75)
Total P (µM)	n.d.	0.57 (0.02-1.27)	1.18 (0.43-8.14)	0.73 (0.11-8)	0.58 (0.29-0.92)
Total N: Total P ratio	n.d.	43.05 (10.2-865)	46.14 (3.1-141.9)	48.46 (3.1-212.5)*	30.6 (12.3-65.7)
DOC (µM)	n.d.	622.5 (361-1589)	431.5 (327-1710)	629.2 (348-1569) **	473.7 (327-1710)

Table S2 Average sea surface temperature and number of days corresponding to cool winter (< 3°C) and warm summer (>16°C) in the Kalmar Sound and the Western Gotland Sea over December 2009-September 2012. Data are from the hydrological model (S-HYPE) available from Swedish Meteorological and Hydrological Institute (SMHI)

Winter				Summer		
Days < 3°C				Days > 16°C		
	2010	2011	2012	2010	2011	2012
Start	30-Dec-09	5-Jan-11	7-Mar-12	30-Jun-10	28-Jun-11	18-Jul-12
Stop	23-Apr-10	17-Apr-11	11-Mar-12	15-Sep-10	13-Sep-11	7-Sep-12
Duration	113	102	4	75	75	49
Mean temp	0.7	0.5	2.8	18.7	18.1	17.4
Min temp	-0.2	-0.3	2.8	16.1	16.1	16.0
Max temp	2.9	2.7	2.9	21.5	20.0	18.8
